# Time-resolved XPCS analysis across broad time-scales using multi-tau two-time correlations

**DOI:** 10.1107/S1600577526002171

**Published:** 2026-03-30

**Authors:** Fabio Brugnara, Marco Cammarata, Yuriy Chushkin, Irene Festi, Massimo Granata, David Hofman, Matteo Leonardi, Daniele Marzi, Denis Nabari, Angela Trapananti, Simone Ziglio, Federico Zontone, Marco Zanatta, Giacomo Baldi

**Affiliations:** a Department of Physics University of Trento Via Sommarive 14 38123Trento Italy; b ESRF – The European Synchrotron, 71 avenue des Martyrs, 38000Grenoble, France; c Laboratoire des Matériaux Avancés – IP2I, CNRS Université Claude Bernard Lyon 1 69100Villeurbanne France; d INFN Trento Institute for Fundamental Physics and Applications 38123Povo Trento Italy; e Physics Division – School of Science and Technology University of Camerino Via Madonna delle Carceri 9 62032Camerino(MC) Italy; f INFN, Sezione di Perugia, Via A. Pascoli, 06123Perugia, Italy

**Keywords:** X-ray photon correlation spectroscopy, multi-tau algorithm, synchrotron data analysis

## Abstract

We present a multi-tau two-time correlation (MT-2TC) analysis for X-ray photon correlation spectroscopy that enables efficient analysis of large-scale, high-frame-rate datasets collected at modern synchrotron beamlines. The method combines the advantages of the multi-tau autocorrelation and two-time correlation approaches, maintaining sensitivity to non-stationary dynamics while reducing computational complexity and memory requirements from quadratic to linear scaling with the number of frames. This formulation not only improves processing efficiency but also extends the accessible correlation time range, allowing analysis over much broader time scales than conventional techniques. The algorithm is implemented in both dense and sparse data representations, allowing direct processing of sparsified photon-counting data from ultra-fast detectors. We demonstrate the performance of the MT-2TC scheme using experiments on a bulk GeO_2_ glass and on a v-Ta_2_O_5_ thin film conducted at the ESRF ID10 beamline with a 1 kHz Eiger 4M detector. The results show perfect agreement with standard correlation analyses while extending the temporal correlation range by nearly two decades and improving signal-to-noise ratios at long lag times. The MT-2TC approach thus provides a scalable and flexible tool for XPCS at fourth-generation synchrotrons, where enhanced coherent flux and detector speed demand correlation strategies capable of spanning microsecond-to-day timescales.

## Abstract 2

A scalable multi-tau two-time correlation framework enables efficient, time-resolved XPCS analysis of long duration high-frame-rate modern synchrotron data while preserving sensitivity to non-stationary dynamics.

## 1. Introduction

X-ray photon correlation spectroscopy (XPCS) is a powerful method for probing slow, nanometre-scale structural dynamics in condensed matter systems. The technique exploits the coherent fraction of synchrotron or free-electron laser X-rays: when a coherent beam is focused onto a sample, the scattered radiation produces a speckle pattern, *i.e.* a granular interference fingerprint of the instantaneous spatial arrangement of scatterers. Recording the temporal fluctuations of this pattern with a fast area detector enables direct measurements of dynamical processes such as diffusion, relaxation, and flow (Sutton, 2008 ▸; Shpyrko, 2014 ▸; Madsen *et al.*, 2016 ▸).

At modern synchrotrons, XPCS takes advantage of the exceptional brightness and coherence of the focused beam. To mitigate radiation damage and to maximize weak scattered signals, pixelated detectors are employed to improve the signal-to-noise ratio (SNR) at fixed dose by averaging over multiple coherence areas (multi-speckle XPCS) (Lumma *et al.*, 2000 ▸; Falus *et al.*, 2006 ▸). The correlation function SNR is given by
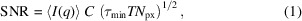
which increases linearly with the average scattered intensity 〈*I*(*q*)〉 and scales with the square root of the number of pixels *N*_px_, the minimum sampling time 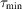, and the measurement time *T*. Here, *C* denotes the contrast, *i.e.* the speckle visibility expected for a fully static sample, and is directly related to the coherence of the X-ray beam (Pecora, 1985 ▸).

Dedicated XPCS beamlines are now standard installations at leading synchrotron facilities worldwide, such as ID10 at ESRF, 8-ID-E at APS, 11-ID at BNL and P10 at PETRA III. Over the past decade, these facilities have seen major developments:

(i) *Coherent flux at fourth-generation synchrotron radiation sources.* Third-generation synchrotron radiation sources typically delivered a coherent flux of 5 × 10^9^ to 5 × 10^10^ photons s^−1^ in the 8–11 keV range [*e.g.* ID10 at ESRF (Favre-Nicolin *et al.*, 2017 ▸), 8-ID-E at APS (Jiang *et al.*, 2023 ▸), P10 at PETRA III (Sprung *et al.*, 2023 ▸)]. The advent of fourth-generation facilities, such as ESRF-EBS and APS-U, has produced a substantial increase in brilliance, and consequently in coherent flux, by up to two orders of magnitude, mainly due to the dramatic reduction of horizontal emittance in the electron beam (Favre-Nicolin *et al.*, 2017 ▸; Raimondi *et al.*, 2023 ▸; Cornet *et al.*, 2024 ▸).

(ii) *Ultra-fast photon-counting pixel array detectors (PADs).* Photon-counting PADs have become the detectors of choice for XPCS thanks to their large dynamic range, negligible readout noise and dark current, and large collection areas (Zhang *et al.*, 2018 ▸). The Eiger 4M is today’s standard detector, offering frame rates up to 4 kHz (DECTRIS AG, 2025 ▸). Recent developments in ultra-fast PADs now enable access to even shorter delay times. Continuous measurements have been demonstrated at 50 kHz for about 50 s and at 1.2 MHz for 4 s in burst mode using the UFXC32k chip (Zhang *et al.*, 2016 ▸; Zhang *et al.*, 2017 ▸) and the related Rigaku XSPA-500k detector (Zhang *et al.*, 2018 ▸). Burst-mode operation of the AGIP detector has reached 195 ns delay times over 700 µs (Jo *et al.*, 2021 ▸), while the TEMPUS detector has recently achieved ∼450 ns in continuous operation using its event-driven mode (Chushkin *et al.*, 2025 ▸).

(iii) *Raw data sparsification.* To handle the huge data rates produced by ultra-fast PADs, raw data sparsification was introduced at the 8-ID-E beamline (Zhang *et al.*, 2021 ▸) and is now standard at ID10 (ESRF), 8-ID-E (APS) and 11-ID (BNL). The approach, motivated by the measured low-count-rate frames of typical XPCS experiments (Chushkin *et al.*, 2012 ▸), stores only non-zero values and their positions, drastically reducing data throughput while preserving the entire measurement information.

(iv) *Beamline stability.* Improvements in mechanical and thermal stability have significantly extended the upper dynamical limit of XPCS. Relaxation times of several hours can now be reliably measured, as shown in slow-dynamics experiments (Ruta *et al.*, 2013 ▸).

The combination of increased coherent flux and ultra-fast PADs dramatically expands the probed range towards faster times. Concurrently, improvements in instrumental stability and data sparsification pipelines enable measurements over hours or even days at high frame rates. For instance, we recently continuously tracked the relaxation dynamics of vitreous GeO_2_ and vitreous Ta_2_O_5_ for up to 16 h with 1 ms resolution, generating over 40 million frames with the Eiger 4M detector at ID10 (see Section 4). These advances allow XPCS to span an unprecedented dynamical window, from microseconds to days. This opens new scientific opportunities, such as capturing both α and β relaxations within a single correlation function, or simultaneously resolving fast and slow dynamics in aging systems. However, these capabilities introduce significant computational challenges, demanding increased processing power and memory for the calculation of correlation functions.

Three distinct approaches are commonly used for data reduction in multi-speckle XPCS: standard intensity correlation (IA) (Lumma *et al.*, 2000 ▸), multi-tau intensity autocorrelation (MT-IA) (Schätzel, 1987 ▸; Cipelletti & Weitz, 1999 ▸), and two-time correlation (2TC) (Sutton *et al.*, 2003 ▸; Bikondoa, 2017 ▸; Cipelletti *et al.*, 2003 ▸). The multi-tau scheme is widely employed for systems where the dynamics do not change significantly during the measurement. It improves the SNR at long delay times and dramatically reduces computational complexity [from *O*(*N*^2^) to 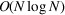] by correlating progressively time-averaged frames. In contrast, the two-time correlation approach is necessary for systems with time-dependent dynamics, such as those driven by external stimuli, undergoing aging, or relaxing toward equilibrium. This method calculates the correlation as a function of both measurement time and delay time, yielding a 2D correlation map.

In this paper, we introduce a new data reduction scheme that combines the advantages of both methods: the *multi-tau two-time correlation* function (MT-2TC). This approach mitigates computational complexity and largely reduces the memory limitations that constrain the number of frames in standard two-time calculations, and provides a more direct quasi-logarithmic visualization of the correlation matrix. Furthermore, the measurement-time average of our function reduces to the classical multi-tau correlation function, allowing for the straightforward recovery of the standard autocorrelation, as in the two-time formalism.

## 2. Multi-speckle correlation functions

### 2.1. Intensity autocorrelation (IA)

Photon correlation spectroscopy with area detectors was first demonstrated in XPCS by Dierker *et al.* (Dierker *et al.*, 1995 ▸) and in dynamic light scattering (DLS) by Kirsch *et al.* (Kirsch *et al.*, 1996 ▸), and later further developed by Cipelletti *et al.* (Cipelletti & Weitz, 1999 ▸) and Lumma *et al.* (Lumma *et al.*, 2000 ▸). In the standard multi-speckle scheme, the intensity autocorrelation (IA) function *g*_2_(*q*, τ) is calculated by averaging over detector pixels with the same scattering vector *q*, 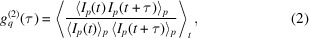
Here, *I*_*p*_(*t*) is the intensity at the pixel *p* and time *t*; the brackets 〈·〉_*p*_ and and 〈·〉_*t*_ denote ensemble (pixel) and time average, respectively. The order of averaging and normalization is important: taking the ensemble average first preserves information on ergodicity (Joosten *et al.*, 1990 ▸), while the chosen normalization suppresses fluctuations in the incident beam (Duri *et al.*, 2005 ▸)^1^.

In a typical XPCS experiment, the detector records a series of speckle patterns (frames) at constant spacing Δ*t*. The data can be arranged as an intensity matrix **I** of dimensions *N*_f_ × *N*_px_ where *N*_f_ is the number of frames and *N*_px_ the number of pixels. When stored as a dense matrix, besides the challenges of data storage, a direct calculation of the numerator in the above expression scales as 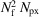, which quickly becomes computationally prohibitive.

As depicted in the *Introduction*[Sec sec1], when detectors operate at high frame rates and scattering signal is low, most entries of **I** are zero. Using sparse storage formats, mainly compressed sparse row (CSR) or column (CSC) representations (Scott & Tůma, 2023 ▸), can drastically reduce the computational cost of evaluating equation (2), since only nonzero elements are processed.^2^ However, row or column indexing in sparse matrices is intrinsically slow, limiting the efficiency of a direct element-wise evaluation of equation (2). A more practical solution is provided by two-time correlation functions exploiting the sparse linear algebra.

### 2.2. Two-time correlation (2TC)

Omitting the time averaging in equation (2), the two-time correlation (2TC) function can be defined as (Sutton *et al.*, 2003 ▸) 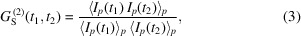
where *t*_1_ and *t*_2_ are the times of the correlated frames. Independently, Cipelletti *et al.* (2003 ▸) introduced an equivalent form, 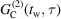, expressed in terms of the waiting time 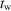 = 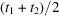 and the lag time τ = |*t*_1_ − *t*_2_|. Using the symmetric definition of the waiting time (Bikondoa, 2017 ▸), 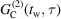 corresponds to a 45° rotation of the 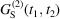 matrix. Within both representations, the standard IA can be computed by time averaging (Duri *et al.*, 2005 ▸) , 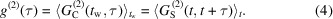
Equations (3) and (4) give a practical computational pathway to compute both 2TC and IA taking advantage of the efficiency of matrix multiplication algorithms,
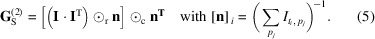
The matrix product **I** · **I**^T^ computes the pixel-averaged correlations in the numerator of equation (3). The subsequent element-wise multiplications, ⊙_r_ (row-wise) and ⊙_c_ (column-wise) with the normalization vector **n** ensures the proper normalization. The entries of the resulting matrix (*N*_f_ × *N*_f_ large) correspond directly to the 2TC: 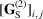 = 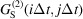.^3^ The IA vector is then obtained by averaging over the offset diagonals of this matrix, *i.e.* averaging over constant lag times: [**g**^(2)^]_*k*_ = 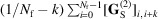.

This matrix-multiplication approach leverages optimized linear algebra routines and is especially powerful for sparse data, where direct indexing is slow. However, it comes at the cost of memory: 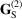 is a dense matrix of dimension *N*_f_ × *N*_f_. For large datasets, this rapidly exceeds available resources (*e.g.*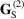 from 1 million frames requires several terabytes). A general workaround is time-binning, where data are coarse-grained to longer frame-times, however, at the expense of losing access to the fastest dynamics. Alternatively, multi-tau autocorrelation offers a way to overcome time-scale limitations.

### 2.3. Multi-tau autocorrelation (MT-IA)

Multi-tau calculation of intensity autocorrelation was first proposed by Schätzle (Schätzel, 1983 ▸) for single area detectors, and later extended to multi-speckle DLS by Cipelletti (Cipelletti & Weitz, 1999 ▸). Instead of following the prescription of equation (2), which requires evaluating all possible frame pairs and thus scales as 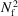, multi-tau algorithms maintain a nearly constant ratio between the delay time τ and the sampling time Δ*t*, still maintaining the temporal resolution 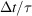

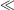
 1. This leads to a quasi-logarithmic sampling of lag times: the correlation function spans several decades of τ with far fewer points, reducing both computational cost and memory requirements, as well as increasing the SNR at large τ as a consequence of the non-constant Δ*t*.

**Figure 1:**
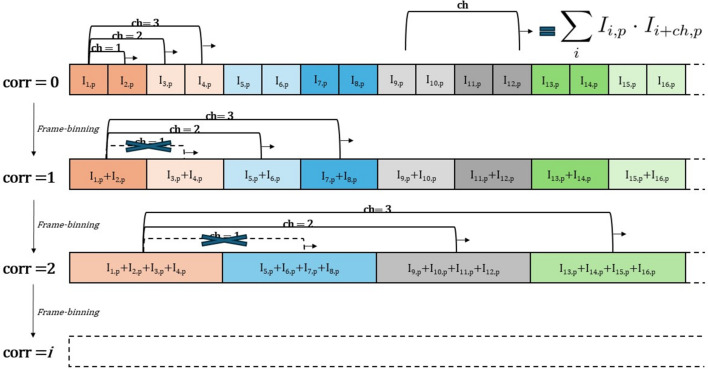
Multi-tau correlator scheme for a single pixel *p* with four channels. Data sampled at Δ*t* are correlated in *corr* = 0 by all channels but the first, then binned and passed to subsequent correlators, each doubling the delay times. Only new lag times (second half of channels) are computed at each stage (Cipelletti & Weitz, 1999 ▸).

Let us first focus on the calculation of the un-normalized IA for a single pixel. The multi-tau algorithm organizes the computation into a cascade of linear correlators (levels), each with a fixed number of channels (four in the example of Fig. 1 ▸).

(i) *First correlator (*corr* = 0)*. The pixel intensity stream, sampled at Δ*t*, is fed into the channels. All channels except the first (which corresponds to zero lag time, *i.e.* the speckle pattern variance) compute correlations at linearly spaced delays τ_*ch*_ = ch*Δ*t*.

(ii) *Subsequent correlators (*corr* = 1, 2,…)*. Before feeding the signal to the next correlator, pairs of consecutive frames are summed, effectively doubling both the sampling time and the accessible delay times. In these correlators, only the second half of the channels are used, since the first half corresponds to lag times already computed at higher temporal resolution.

(iii) *Iteration*. This binning–correlation process is iteratively repeated, each stage doubling the frame time and extending the correlation function to longer τ. The multi-tau autocorrelation (MT-IA) function is obtained by merging the outputs of all correlators.

Finally, the MT-IA functions calculated for all pixels with the same scattering wavevector are averaged and normalized.^4^ Multi-speckle multi-tau autocorrelation is the starting point of our novel correlation scheme.

## 3. Multi-tau two-time correlation (MT-2TC)

The 2TC matrix defined in equation (5) is a standard tool in XPCS data analysis. It provides information on sample ergodicity and stationarity, allows verification of measurement stability, and highlights temporally correlated noise sources (Bikondoa, 2017 ▸) (*e.g.* spurious high-count signals from environmental radioactivity appearing in consecutive frames). Once stationarity is established, the IA is obtained by time-averaging over the selected stationary time ranges. As already noted, the main limitation of this method lies in the size of the 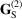 matrix. In addition, when the measurement time far exceeds the sample decorrelation time, visualizing the entire matrix becomes uninformative. In such cases, data are either inspected through sub-matrices or, as in recent studies (Perakis *et al.*, 2017 ▸; Chèvremont *et al.*, 2024 ▸), reformulated in the 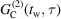 representation and plotted with a logarithmic τ axis. To overcome these limitations, we propose a multi-tau definition of the 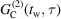 function, quasi-logarithmically spaced in τ. This approach extends the standard multi-tau scheme of Fig. 1 ▸ by avoiding temporal averaging.

We denote frame-shifts as follows: **I**[*i*:] is the sub-matrix of **I** starting from row *i*, and **I**[: −*j*] excludes the last *j* rows. Frame binning for the *corr*-th correlator is defined as 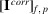 = 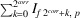.^5^ The non-normalized multi-tau 2TC is then defined as a set of vectors of different sizes, one for each correlator index *corr* = 0, 1,… and channel index *ch* = 0, 1,…, *N*_*ch*_ − 1: 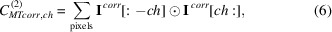
where ⊙ is the element-wise (Hadamard) product. For each correlator and channel, the intensity matrix is first binned by 2^*corr*^, then element-wise multiplied with its frame-shifted copy (by *ch* rows), and finally summed over pixels. Normalization follows equation (3), 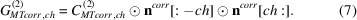
As in the standard multi-tau scheme, the correlator and channel indices determine the lag time τ, while the *i*th element of the vector 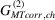 corresponds to a waiting time *t*_w_,
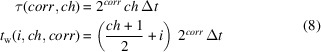
Computationally, each Hadamard product in the denominator requires roughly *N*_px_*N*_f_/2^*corr*^ multiplications per correlator and channel. Computing the full object thus involves about 2*N*_px_*N*_*ch*_*N*_f_ operations.^6^ Similarly, the number of elements in 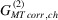 scales as 2*N*_f_*N*_*ch*_. For large datasets, both the computational cost and storage requirements are therefore reduced from quadratic to linear in *N*_f_. This enables calculation and storage of 2TC functions irrespective of frame rates and measurement times, thus allowing operation at the full detector speed. Visualization is naturally achieved in a *t*_w_–τ plot with logarithmic τ, which conveniently displays the full dynamics of the sample—even when relaxation times are much shorter than the total acquisition time—and facilitates verification of stationary ranges.

**Figure 2:**
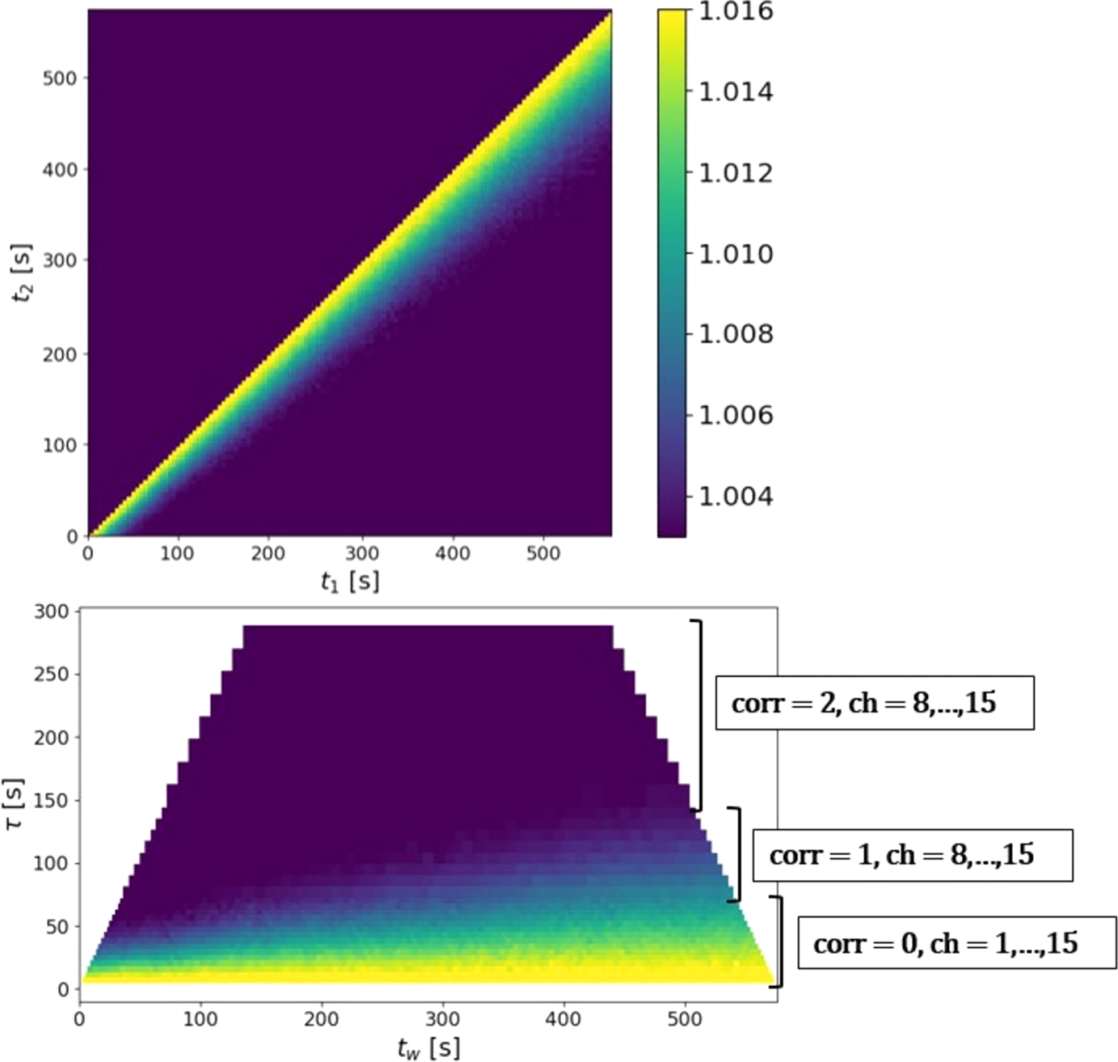
Comparison of the standard 2TC (top) and MT-2TC (bottom) computed for a v-Ta_2_O_5_ measurement at a low frame rate (Δ*t* ≃ 4 s), chosen to improve the SNR (see Section 4.2 for details). The labels indicate which correlator and which set of channels correspond to the rows of the MT-2TC. Notably, the first correlator consists of 15 channels (the first channel is omitted, as it corresponds to the self-correlation of frames), while the other two correlators consist of eight filled channels each.

As an example, Fig. 2 ▸ compares the standard 2TC (top) with the MT-2TC (bottom), computed on a v-Ta_2_O_5_ 128 frames measurement at low frame rate Δ*t* ≃ 4 s (details given in Section 4.2). In the multi-tau calculation, we used 16 channels, which is sufficient to avoid time-blurring effects as, apart from the first half of the first correlator’s channels, they always keep 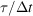 > 8. The MT-2TC can be viewed as a 45° rotation of the 2TC matrix, but with the lag time τ quasi-logarithmically sampled: the rectangle rows corresponding to each correlator and channel double in both height and width every eight rows (except for the first correlator, which has 16 channels). In this example, both the 2TC and the MT-2TC reveal the same physical information, namely the sample dynamics evolving with an increasing relaxation time. However, important practical differences arise:

(i) Although both are computed from the same 128 frames, they contain very different numbers of elements. The 2TC has 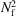 = 16384 entries, while the MT-2TC contains fewer than 2*n*_*ch*_*N*_f_ = 4096 entries. This gap grows quadratically with *N*_f_.

(ii) To perform this comparison, we simulated 4 s frames by binning the original 1 ms data, so that the standard 2TC achieves a reasonable SNR. At much higher frame rates the 2TC would become unusable, dominated by noise. By contrast, the MT-2TC remains robust: for each lag time it shows the same SNR irrespective of the frame rate, since its SNR naturally improves with increasing τ.

Finally, the IA function, consistent with that of Section 2.1, can be recovered by averaging over *t*_w_, and errors can be computed accordingly,^7^
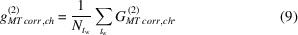


### 3.1. MT-2TC from sparse matrices

In principle, equation (7) enables the calculation of both IA and 2TC for arbitrarily large *N*_f_. In high-frame-rate and long-duration measurements, however, data are typically stored in sparse formats to circumvent memory limitations. Therefore, an efficient implementation of equation (7) must explicitly operate on sparse data structures. A key difficulty arises in the binning step: binning sparse intensity matrices is highly inefficient, as it neither reduces memory usage nor simplifies subsequent calculations. In fact, binning a sparse matrix by a factor of 2 usually results only in a frame re-indexing of the events, since two consecutive frames rarely record photons in the same pixel. To address this, we propose a two-step implementation of equation (7) that fully advantage of the sparse formalism.

#### 3.1.1. Step 1: sparse-domain 2TC sub-matrices

**Figure 3:**
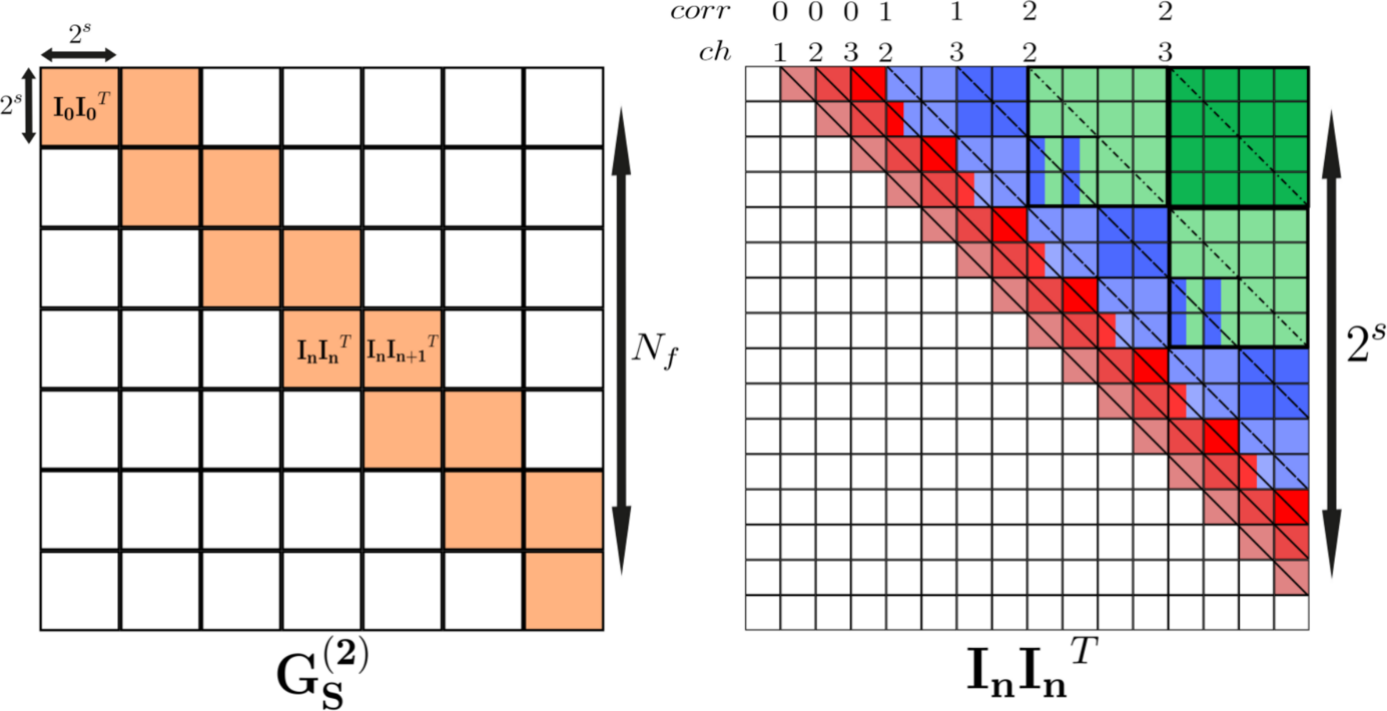
Illustration of the two-step sparse implementation of the multi-tau 2TC. Left: decomposition of the full 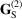 into diagonal and off-diagonal sub-matrices computed from sparse blocks of **I**. Right: mapping of a 2TC sub-matrix into the multi-tau representation via successive binning and extraction of *ch*-offset diagonals. In this example, *s* = 4 and four channels are used. Double-colored entries indicate values used in different multi-tau correlators.

We first compute the linear 2TC matrices using equation (5), but restricted to small sub-matrices of **I**. These are defined by a parameter *s* as **I**_*n*_ = **I**[*n*2^*s*^:(*n* + 1)2^*s*^] with *n* = 0, 1,…,*N*_f_/2^*s*^. For each *n*, two consecutive 2TCs are calculated: (i) the autocorrelation of **I**_*n*_, and (ii) the cross-correlation of **I**_*n*_ with **I**_*n*+1_.^8^ These correspond to the diagonal blocks of the entire 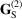 matrix (Fig. 3 ▸, left). Each 2TC sub-matrix is then converted into its multi-tau version, subsequently filling the first correlators of 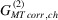 by appending the correlation values at the end of its vectors. For each correlator, this conversion is achieved by binning the 2TC sub-matrices, both by rows and columns, by 2^*corr*^, and extracting the channel vectors from the corresponding *ch*-offset diagonals (Fig. 3 ▸, right). The cross-correlation matrices **I**_*n*_**I**_*n*+1_ are treated analogously, except that their first correlators and channels start from the corner rather than from the diagonal. It can be shown that this algorithm fills the first correlators equivalently to equation (7), but moves time-binning after the correlation computation. The advantage of this approach is that it avoids explicit frame binning, still avoiding high memory consumption, since only two small **I** sub-matrices are processed at each step, which are then transformed from the linear 2TC into its multi-tau representation.

#### 3.1.2. Step 2: dense-domain computation

The second stage follows equation (7). The intensity matrix **I** is time-binned by a factor 2^*s*+1^ and converted into a standard dense matrix. From this point, subsequent correlators are computed via the Hadamard product and binning scheme of equation (7). In practice, the optimal choice of *s* is such that 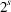 ≃ 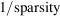, *i.e.* binning becomes advantageous once the resulting matrix is effectively dense.

### 3.2. Implementation and memory considerations

As described in Section 3.1, the MT-2TC calculation for sparse data is divided into two main stages. In the first stage, standard two-time correlation (2TC) sub-matrices are computed from successive batches of 2^*s*^ frames. At this stage, only a limited number of intermediate quantities are required. Two arrays, *I*_0_ and *I*_1_ (each of dimension 2^*s*^ × *N*_px_), store the frame batches used to compute diagonal and off-diagonal 2TC sub-matrices. The corresponding correlation results are stored in an intermediate matrix 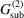 of dimension 2^*s*^ × 2^*s*^. The output of this stage consists of (i) a time-binned version of the original frame sequence, denoted *I*_dense_ (of dimension *N*_f_/2^*s*^ × *N*_px_), and (ii) a partially filled MT-2TC object, in which only the channels associated with the first correlators are populated.

The first-stage computation is performed by iterating over the dataset in steps of 2^*s*^ frames. At each iteration, two consecutive frame batches, *I*_0_ and *I*_1_, are loaded into memory. These batches are used to compute both the diagonal and off-diagonal two-time correlation (2TC) sub-matrices, which are temporarily stored in the intermediate array 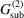. The resulting sub-matrices are then transformed into their multi-tau representation and accumulated in the corresponding channels of the MT-2TC object. Finally, the frames *I*_0_ are time-binned by summation to produce the corresponding dense frame, which is stored in the vector *I*_dense_ for subsequent processing in the dense stage of the algorithm.

In the second stage, the MT-2TC is completed by operating on the dense (time-binned) frames. This step involves matrix slicing and dense matrix elementwise multiplications, followed by successive binning of *I*_dense_ by a factor of two at each iteration, as described by equations (6) and (7).

In the present implementation, the computation of sparse 2TC sub-matrices relies on Intel MKL sparse matrix-multiplication routines (Intel Corporation, 2023 ▸), while the frame-binning step and all dense operations are implemented using the *NumPy* library (Harris *et al.*, 2020 ▸).

#### 3.2.1. Computational time

The computational time of the MT-2TC algorithm scales linearly with the number of frames *N*_f_. In the first stage, the number of loop iterations scales as *N*_f_/2^*s*^. In the second stage, the initial number of dense frames to be processed is also*N*_f_/2^*s*^, and the first iteration dominates the computational cost, since subsequent iterations operate on progressively halved frame sequences. As a result, the total computational time increases approximately linearly with *N*_f_.

This behavior contrasts with the standard 2TC approach, whose computational cost and memory usage scale quadratically with the number of frames. However, the standard matrix-multiplication-based 2TC benefits from highly optimized linear algebra routines and remains faster until memory limitations come into play. As *N*_f_ increases and the full 2TC matrix can no longer be stored in memory, the standard approach becomes impractical. In this regime, the MT-2TC retains linear scaling with *N*_f_, providing a decisive advantage in terms of feasibility for large datasets.

#### 3.2.2. Memory considerations

The main advantage of the MT-2TC method is the strong reduction of memory usage. In standard 2TC calculations, the dominant memory limitation arises from storing the full 2TC matrix, whose size scales as *N*_f_ × *N*_f_. For example, on typical beamline computing infrastructure such as that available at the ESRF, storing the full 2TC matrix for a dataset of 200000 frames using 32-bit floating-point numbers would require approximately 160 GB of memory, already at the limits of workstations equipped with a few hundred gigabytes of RAM.

In contrast, the MT-2TC correlation object scales linearly as 2 × *N*_f_ × *N*_ch_. For the same dataset and using 16 channels, the MT-2TC object occupies only about 25.6 MB. This directly addresses the dominant memory bottleneck associated with the size of the two-time correlation matrix in large-frame XPCS datasets.

**Figure 4:**
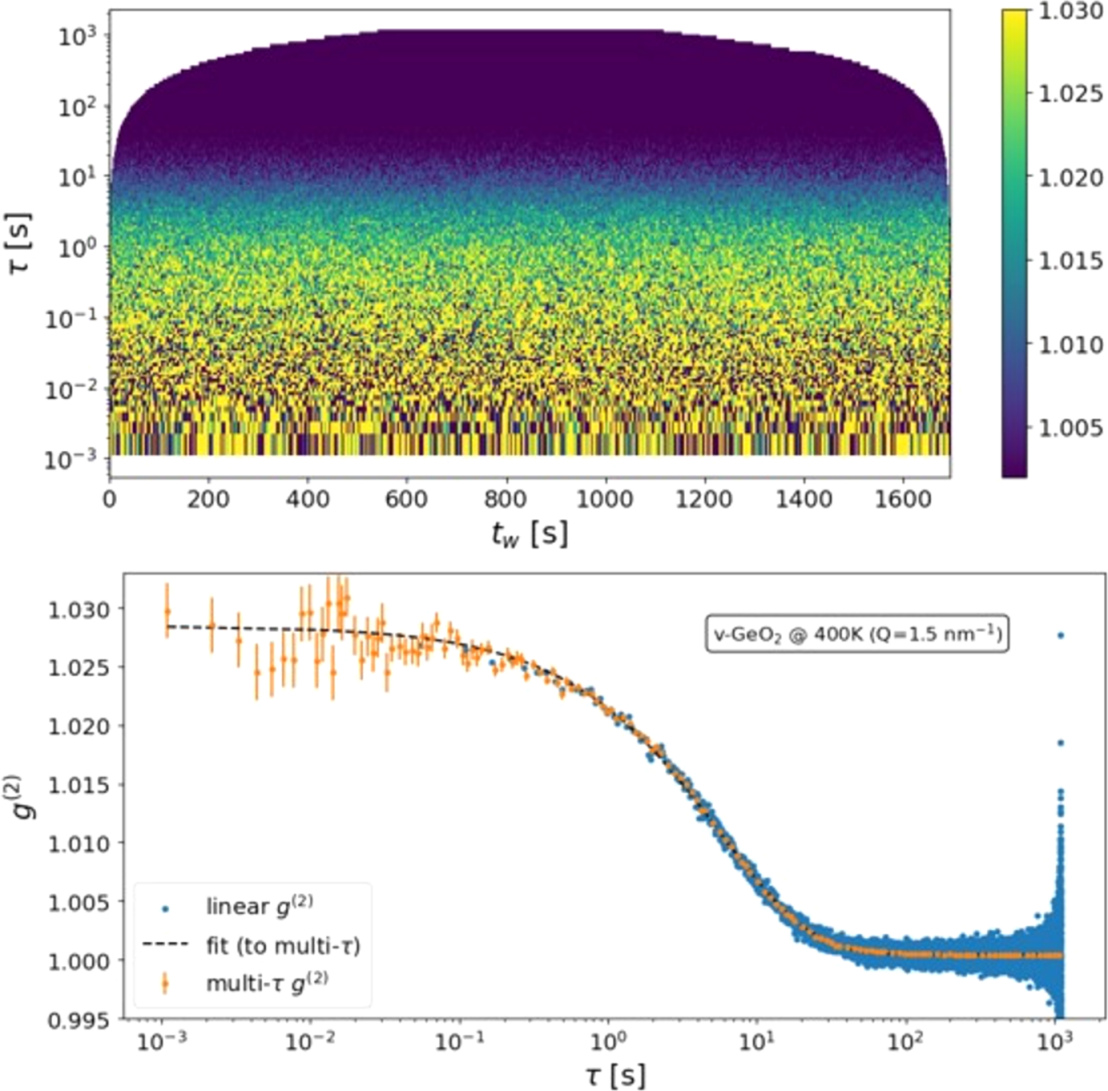
MT-2TC (top) and IA (bottom) computed on X-ray-damaged v-GeO_2_ at 400 K, using 16 channels to preserve temporal resolution. Top: two-time correlation function on 1000000 frames at 1 ms, plotted with lag time on a logarithmic axis, allowing visualization of the relaxation process across the entire measurement window. Bottom: comparison of the multi-tau IA (orange error bars) with the standard linear definition of equation (2) (blue dots), the latter computed from 20000 frames at 50 ms to avoid memory limitations.

Additional memory is required for intermediate quantities. The sparse frame batches *I*_0_ and *I*_1_ each contain of the order of *N*_px_ non-zero values and therefore require limited memory.^9^ The largest intermediate objects are the correlation sub-matrix 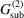 and the dense, time-binned frame sequence *I*_dense_, whose sizes depend on the choice of the parameter *s*. For the example shown in Fig. 4 ▸ (top), the optimal choice is *s* = 14. In this case, computing the MT-2TC for 1 million frames requires approximately 1 GB of memory for each of 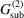 and *I*_dense_. Given the number of frames involved, a total memory requirement of a few gigabytes represents a substantial reduction compared with standard two-time correlation calculations.

Importantly, the algorithm does not require the full dataset to be loaded into memory at once and therefore supports chunked processing, in which data are handled in successive time blocks. At any given time, only the frame batches *I*_0_ and *I*_1_ need to reside in memory. As a result, the method supports datasets that approach or exceed available RAM, with practical limitations progressively determined by the speed of data loading from disk and computation, rather than by memory capacity.

## 4. Experimental demonstrations and perspectives

From a practical standpoint, the MT-2TC approach is particularly well suited to high-frame-rate XPCS experiments involving large numbers of frames. By preserving access to the full two-time information while drastically reducing memory requirements, it enables efficient storage and visualization, as well as flexible *a posteriori* selection of time windows without recomputing correlations, simplifying both online inspection and post-processing of large datasets.

In this section, we illustrate the versatility and effectiveness of the proposed tool through two recent experiments we carried out at the ID10 beamline of ESRF using the Eiger 4M detector working at a frame rate of 1 kHz. The first case concerns the relaxation dynamics of v-GeO_2_, where our aim is to study the temperature dependence of the contrast and its connection to the non-ergodicity of the glass. The second example addresses X-ray-induced dynamics in a thin film of v-Ta_2_O_5_, obtained by ion beam sputtering, comparing as-deposited and annealed samples. Here, the focus was on relaxation processes occurring on the millisecond timescale, which are particularly relevant because they dominate the noise response of ground-based gravitational-wave detectors, such as Advanced Virgo (Acernese *et al.*, 2014 ▸), Advanced LIGO (Aasi *et al.*, 2015 ▸) and KAGRA (Aso *et al.*, 2013 ▸), at frequencies in the range between 10 and 1000 Hz (Steinlechner, 2018 ▸).

For both oxide samples, the dynamics observed at temperatures below the glass transition are dominated by X-ray-induced processes, *i.e.* relaxations activated by the interaction of the intense coherent X-ray beam with the sample, primarily through bond breaking due to the radiolysis process, which superimpose on the intrinsic material dynamics (Ruta *et al.*, 2017 ▸; Martinelli *et al.*, 2023 ▸).

### 4.1. XPCS at ms time resolution on v-GeO_2_

The experiment was performed in the small-angle configuration of the ID10 beamline: the detector was centered at 1.75° using a 9.7 keV X-ray beam, giving access to the 1.2–2.2 nm^−1^*Q*-range. To slow down the X-ray-induced dynamics such that it fell within our measurement time window, the 1.16 × 10^12^ photons s^−1^ beam was defocused to 30 µm × 40 µm on the ∼100 µm-thick v-GeO_2_ slab sample. XPCS scans were collected at a 1 kHz frame rate for 15 temperatures in the 300–1100 K range. The samples were prepared starting from a bulk cylinder of v-GeO_2_, obtained with the standard melt-quenching method. The cylinder was cut into small slices and thinned with polishing paper, reaching 100 ± 10 µm of thickness. The X-ray-induced relaxation time never exceeds 6 s; however, because of the very low scattered signal (∼0.2 photons pixel^−1^ s^−1^), we took ∼1 h scans exploiting the stationary dynamics of the sample to improve the SNR (Cipelletti & Weitz, 1999 ▸). Regardless of temperature (below *T*_g_), the sample initially displayed non-stationary dynamics: during the first ∼200 s of beam exposure the relaxation slows down, from millisecond to second time scales. After this transient regime, the dynamics became stationary. In the experiment, we investigated the dynamics of this ‘damaged’ (or ‘rejuvenated’) glass (Baglioni *et al.*, 2024 ▸; Alfinelli *et al.*, 2023 ▸), thus measuring on the same irradiated spot, within the regime of stationary dynamics.

The stationarity of the dynamics is quantified using the novel MT-2TC. An example, computed for the 400 K scan at *Q* = 1.7 ± 0.1 nm^−1^ (1000000 frames at 1 ms), is shown in Fig. 4 ▸. The two-time correlation function, naturally represented with the lag time on a logarithmic scale, allows the relaxation process to be monitored across the entire measurement time, even when the relaxation itself is much shorter than the measurement time. As expected, correlations at shorter lag times are noisier due to the limited number of correlated photons, while at longer lag times the statistics improve and averaging is no longer needed to obtain stable values. Once stationarity is established, the *g*^(2)^ function is obtained by averaging over the rows of the 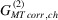 object, as shown in the lower panel of Fig. 4 ▸. Here, the agreement with the Kohlrausch–Williams–Watts (KWW) relaxation function over six decades in time is significant.

The multi-tau IA is compared with the standard linear definition of equation (2), computed from 20000 frames obtained by binning the original 1 million frames by a factor of 50 in order to avoid memory limitations. Otherwise, when using 32-bit floating-point numbers, the full 2TC matrix would require approximately 4 TB of memory. The two methods give consistent results, but the multi-tau calculation provides a significant advantage in terms of SNR, especially at long lag times, and, more importantly, extends the accessible time window by almost two decades. Given our experimental objectives, reliable access to the low-lag-time regime, *i.e.* lag times much shorter than the X-ray-induced relaxation time, is essential to resolve the plateau before the initial decay of the correlation function and thus to accurately determine the contrast, which would otherwise be compromised by excessive frame binning. A detailed analysis of the studied temperature dependence of the non-ergodicity factor will be presented in a future publication.

### 4.2. X-ray induced relaxation dynamics in v-Ta_2_O_5_

The experiment was carried out in the wide-angle configuration of the ID10 beamline, with the detector angle varied between 1.5° and 10° to probe the *Q*-dependence of the induced relaxation within the range 3–20 nm^−1^, using a working energy of 21.7 keV. The samples consisted of two 2.5 µm-thick v-Ta_2_O_5_ films deposited on Si substrates by ion-beam sputtering at the Laboratoire des Matériaux Avancés (LMA): one in the as-deposited state and the other annealed at 500°C for 10 h (additional details on sample preparation will be provided in a forthcoming publication, as well as a discussion on the XPCS results). The 0.8 × 10^12^ photons s^−1^X-ray beam was tightly focused to a spot size of 10 µm × 7 µm in order to increase the dose rate and accelerate the beam-induced dynamics. The experiment is performed in transmission geometry through the 0.5 mm Si substrate, which is almost transparent at this working energy.

**Figure 5:**
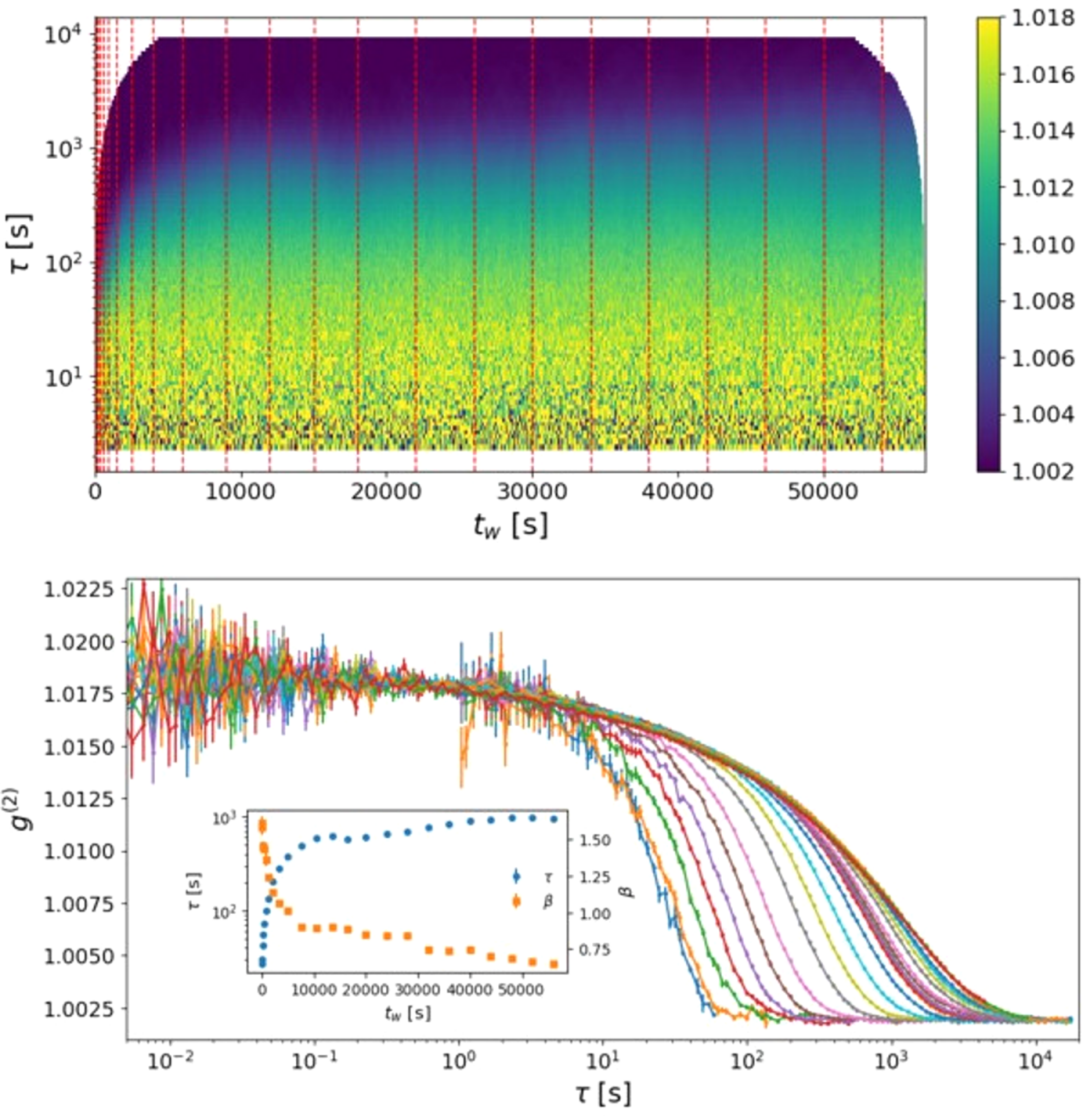
X-ray-induced relaxation dynamics of the as-deposited v-Ta_2_O_5_ sample at *Q* = 20 nm^−1^. Top: MT-2TC function for lag times τ > 1 s, computed from 60000000 frames at 1 ms, capturing the evolution of the relaxation dynamics during beam exposure. Vertical dashed red lines indicate the stationary time windows selected for IA analysis. Bottom: corresponding *g*^(2)^ functions obtained by averaging over the selected windows. Access to faster dynamics is limited to the wider time windows, which exhibit improved SNR; therefore, only *g*^(2)^ from these windows are shown for short lag times (τ < 1 s). Inset: extracted relaxation times and stretching exponents, which progressively increase and decrease, respectively, with dose, both following an approximately exponential trend.

Subjected to such X-ray dose, the relaxation dynamics evolves on the scale of a day, requiring measurements to be monitored over several hours. Fig. 5 ▸ shows this evolution for the as-deposited sample at *Q* = 20 nm^−1^. The MT-2TC plot (top), computed from 60000000 frames at 1 ms, captures the full evolution of the dynamics across the measurement window. The characteristic relaxation time starts at a few tens of seconds and rapidly increases to thousands of seconds, and the relaxation function progressively stretches, evidencing the heterogeneous nature of the sample dynamics. The MT-2TC representation also guides the choice of stationary time-windows for computing autocorrelation functions and extracting KWW relaxation parameters. In practice, it suggests exponentially increasing window widths during the first hours, followed by equally large windows at later times (vertical red dashed lines). The *g*^(2)^ functions are then obtained by *t*_w_-averaging over the appropriate segments of the MT-2TC [according to equation (8)], ensuring that the averaged correlations are calculated from frames belonging to the selected stationary windows.

From a practical perspective, the MT-2TC for this dataset, comprising 60 million frames, was computed only once and required approximately 30 min on a standard analysis workstation. Subsequent calculations of the *g*^(2)^ functions for different waiting times and window selections involve only cuts and averages of the precomputed MT-2TC, and therefore incur negligible additional computational cost. This illustrates a key practical advantage of the method for long-duration experiments, where repeated recalculation of two-time correlations would otherwise be prohibitive.

Fig. 5 ▸ (bottom) shows the resulting *g*^(2)^ functions calculated for the selected time windows. As expected from equation (1), the SNR improves with increasing window width. Consequently, access to faster dynamics is restricted to the wider time windows, since the correlations computed over shorter intervals are dominated by statistical noise. The fitted *t*_w_ dependence of the relaxation time τ and the stretching exponent β is presented in the inset. For the as-deposited sample, the relaxation time progressively increases with exposure, following an approximately exponential trend.

We note that, when the induced decorrelation becomes very long, in contrast to the v-GeO_2_ measurements, the relaxation over the full dynamical range cannot be adequately described by a single KWW function. In particular, in the fast lag-time range between 10 ms and 10 s, the correlation function exhibits a slow decay that precedes the main relaxation and cannot be captured by a stretched exponential form. We are currently investigating whether this behavior is indicative of the presence of a secondary relaxation process, possibly related to the thermal Brownian noise in gravitational waves detectors. Access to this extended time window is therefore essential to correctly characterize the dynamics and to avoid oversimplified fits based on a restricted lag-time range.

### 4.3. Further potential applications of the MT-2TC

Beyond the experimental demonstrations presented above, the MT-2TC approach is expected to be broadly applicable to XPCS measurements involving large datasets, high frame rates, and time-evolving dynamics. By providing access to the full two-time information while drastically reducing data size, the method enables flexible *a posteriori* analysis and efficient handling of long-duration measurements.

More generally, one of the most promising applications of the MT-2TC is the detection of two-step relaxation processes in glassy systems, such as the coexistence of α and β relaxations. While such phenomena are well known in light scattering and XPCS studies (Rössler & Becher, 2025 ▸), they have not yet been clearly resolved with X-rays in the regimes targeted here. Access to correlation functions over lag-time ranges significantly wider than the characteristic decorrelation region is essential to identify these behaviors and to avoid masking slow or intermediate relaxation components.

In addition, the MT-2TC representation is particularly valuable for experiments exhibiting non-stationary or heterogeneous dynamics, where the identification of stationary time windows is nontrivial. In these cases, it provides an efficient and intuitive way to monitor the temporal evolution of the dynamics and to guide the selection of appropriate averaging intervals, especially in dose-dependent or aging experiments.

In practice, the advantages of the MT-2TC become most apparent when large numbers of frames must be handled simultaneously. While for small datasets the standard matrix-multiplication-based two-time correlation remain faster due to highly optimized linear algebra libraries, this situation changes as soon as memory limitations prevent computing and storing the full *N*_f_ × *N*_f_ correlation matrix. The MT-2TC is specifically designed to operate in this regime, which is increasingly common in high-frame-rate XPCS experiments involving long acquisition times and evolving dynamics.

## 5. Conclusions

The multi-tau two-time correlation (MT-2TC) algorithm presented here constitutes a significant advance in the computational analysis of XPCS data. By integrating the multi-tau scheme into the two-time correlation formalism, the method preserves sensitivity to non-stationary and time-evolving dynamics while drastically reducing computational and memory demands. Its quasi-logarithmic sampling in lag time enables the computation of correlation functions over very wide temporal ranges, providing natural visualization of dynamical processes spanning many orders of magnitude in time. Moreover, the MT-2TC matrix can be computed once and stored, allowing subsequent analyses, such as stationary time window selection, averaging and fitting, to be performed flexibly without recomputation. The sparse-domain implementation further ensures compatibility with modern fast photon-counting detectors and event-based data streams.

Applied to experimental data from vitreous GeO_2_ and Ta_2_O_5_, the MT-2TC approach yields correlation functions consistent with conventional methods but with improved SNRs and extended dynamical coverage. The technique enables reliable identification of ergodic intervals and efficient extraction of relaxation parameters, even for measurements exceeding millions of frames.

The MT-2TC framework is therefore particularly well suited for upcoming fourth-generation synchrotron sources, where increased coherent flux and detector bandwidth can produce data volumes beyond the limits of traditional correlation algorithms. By combining computational scalability and full temporal sensitivity, the method provides a robust and flexible tool for exploring both stationary and time-dependent phenomena across broad dynamical ranges.

## 6. Code availability

The implementation of the multi-tau two-time correlation method presented in this work is publicly available under an open-source license at https://github.com/FabioBrugnara/XPCS_library.

## Footnotes

1 Different definitions of *g*_2_(*q*, τ) exist in the literature, varying in both the order of averaging and the choice of normalization (Cipelletti & Weitz, 1999 ▸; Lumma *et al.*, 2000 ▸; Duri *et al.*, 2005 ▸; Chushkin *et al.*, 2012 ▸; Bikondoa, 2017 ▸).

2 A related approach based on event correlators was proposed by Chushkin *et al.* (2012 ▸), showing dramatic speed-ups.

3 Since  is symmetric, only one half of the matrix needs to be computed.

4 The autocorrelation functions are first averaged over pixels, and later normalized. This order is essential for non-ergodic samples, since it directly yields the ensemble-averaged IA function (Cipelletti & Weitz, 1999 ▸).

5 The last frames are discarded if 2^*corr*^ is not a divisor of *N*_f_.

6 This estimate follows a geometric series and overestimates the true number of multiplications.

7 When averaging over a restricted observation-time range, care must be taken to truncate at the appropriate correlator index, since higher-order correlators may correlate frames lying outside the chosen *t*_w_ interval under the symmetric definition.

8 The extension of equation (5) to this case replaces the transposed **I**_*n*_ matrix with **I**_*n*+1_. The same happens in the normalization.

9 Here we assume the optimal choice *s* ≃ , as discussed previously.
